# Fertilization Following Pollination Predominantly Decreases Phytocannabinoids Accumulation and Alters the Accumulation of Terpenoids in Cannabis Inflorescences

**DOI:** 10.3389/fpls.2021.753847

**Published:** 2021-11-05

**Authors:** Carni Lipson Feder, Oded Cohen, Anna Shapira, Itay Katzir, Reut Peer, Ohad Guberman, Shiri Procaccia, Paula Berman, Moshe Flaishman, David Meiri

**Affiliations:** ^1^The Laboratory of Cancer Biology and Cannabinoid Research, Faculty of Biology, Technion-Israel Institute of Technology, Haifa, Israel; ^2^Agricultural Research Organization (ARO), Volcani Center, Institute of Plant Sciences, Rishon LeZion, Israel

**Keywords:** *Cannabis*, cannabinoids, terpenoids, secondary metabolites, chromatography/mass spectrometry, analytical—methods, gas chromatography, high pressure liquid chromatography

## Abstract

In the last decades, growing evidence showed the therapeutic capabilities of *Cannabis* plants. These capabilities were attributed to the specialized secondary metabolites stored in the glandular trichomes of female inflorescences, mainly phytocannabinoids and terpenoids. The accumulation of the metabolites in the flower is versatile and influenced by a largely unknown regulation system, attributed to genetic, developmental and environmental factors. As *Cannabis* is a dioecious plant, one main factor is fertilization after successful pollination. Fertilized flowers are considerably less potent, likely due to changes in the contents of phytocannabinoids and terpenoids; therefore, this study examined the effect of fertilization on metabolite composition by crossbreeding (-)-Δ^9^-*trans*-tetrahydrocannabinol (THC)- or cannabidiol (CBD)-rich female plants with different male plants: THC-rich, CBD-rich, or the original female plant induced to develop male pollen sacs. We used advanced analytical methods to assess the phytocannabinoids and terpenoids content, including a newly developed semi-quantitative analysis for terpenoids without analytical standards. We found that fertilization significantly decreased phytocannabinoids content. For terpenoids, the subgroup of monoterpenoids had similar trends to the phytocannabinoids, proposing both are commonly regulated in the plant. The sesquiterpenoids remained unchanged in the THC-rich female and had a trend of decrease in the CBD-rich female. Additionally, specific phytocannabinoids and terpenoids showed an uncommon increase in concentration followed by fertilization with particular male plants. Our results demonstrate that although the profile of phytocannabinoids and their relative ratios were kept, fertilization substantially decreased the concentration of nearly all phytocannabinoids in the plant regardless of the type of fertilizing male. Our findings may point to the functional roles of secondary metabolites in *Cannabis*.

## Summary

Fertilization of Cannabis decreases phytocannabinoids accumulation and alters the accumulation of terpenoids from distinct families.

## Introduction

*Cannabis sativa* L. (*Cannabis*) has been known as a medicinal plant since ancient times (Bonini et al., 2018). During the last two decades, many studies added to the growing evidence for its therapeutic effects in a wide range of conditions such as neurodegenerative disorders (Fernández-Ruiz, 2019; Cassano et al., 2020), pain (Starowicz and Finn, 2017), epilepsy (Franco et al., 2021), multiple sclerosis (Rice and Cameron, 2017), and others (for review, see Gonçalves et al., 2019). These therapeutic abilities have been attributed to the secondary metabolites biosynthesized in *Cannabis* (Andre et al., 2016). Around 500 different secondary metabolites have been identified (ElSohly and Slade, 2005; Flores-Sanchez and Verpoorte, 2008). These belong to several groups of compounds including phytocannabinoids, terpenoids and flavonoids. The most characterized to date are the phytocannabinoids, lipophilic compounds made of isoprene units (five-carbon building blocks) (Hanuš et al., 2016), which are almost exclusive to *Cannabis* (Gülck and Møller, 2020). More than 140 different phytocannabinoids are accumulated to various extents in glandular trichomes that are located in the aerial parts of the plant and mostly on the female flowers, which are arranged in a cluster on the stem of the inflorescence (Hanuš et al., 2016). The phytocannabinoids can be classified into several subclasses according to their chemical structures, including the (-)-Δ^9^-*trans*-tetrahydrocannabinol (THC) and cannabidiol (CBD) families as well as cannabinol (CBN), cannabigerol (CBG), cannabichromene (CBC), and others (Flores-Sanchez and Verpoorte, 2008; Berman et al., 2018). A second large group of metabolites is the terpenoids, which are also found in many other plants. These metabolites are closely related to phytocannabinoids, sharing the same isoprenoid precursor and built up by branched isoprene units (Booth et al., 2020). Terpenoids are responsible for the fragrance and taste of the plant and are suggested to also have defensive roles. They also contribute to the therapeutic effects attributed to *Cannabis* (Russo and Marcu, 2017). Another group of metabolites worth mentioning is flavonoids. Among this group, which is widespread in the plant kingdom, there are three specific prenylated flavonoids, termed Cannflavins A–C, which are unique to *Cannabis* and show potent anti-inflammatory abilities (Radwan et al., 2008; Rea et al., 2019; Erridge et al., 2020).

Ongoing research is focused on matching specific metabolites found in the plant and their therapeutic capabilities. To this end, specialized analytical methods have been developed in order to obtain precise knowledge on all the components of the plant and the effects they are responsible for. Currently, more than 90 phytocannabinoids and 100 terpenoids are routinely identified and quantified to obtain an overall chemical profile of each chemovar used for a medicinal purpose (Berman et al., 2018; Shapira et al., 2019). In parallel to the search for specific biological activities of the secondary metabolites, broad ongoing research is focused upon the elucidation of *in planta* metabolites’ biosynthesis, transport and accumulation pathways. Genome, transcriptome and proteome data have been published since 2011 (van Bakel et al., 2011; Laverty et al., 2019; Vincent et al., 2019; Livingston et al., 2020; McGarvey et al., 2020), and have been integrated into a genomic database for *Cannabis* (CannabisGDB) (Cai et al., 2021). Biosynthetic pathways are being unraveled, and recently more than 30 *Cannabis* specific terpenoid synthases have been characterized (Booth et al., 2017, 2020; Allen et al., 2019; Zager et al., 2019; Livingston et al., 2020). In addition, the environmental and developmental factors that affect metabolite accumulation are also studied, such as light (Hawley et al., 2018; Magagnini et al., 2018; Namdar et al., 2019), soil and harvest time (Meier and Mediavilla, 1998; Bernstein et al., 2019; Chandra et al., 2020). The increasing information on the impact of these different factors on metabolite accumulation has the prospect of developing specific chemovars harboring a pre-planned group of metabolites (Romero et al., 2020).

This study examined the effect of an additional factor, the fertilization of *Cannabis* flowers following pollination of the pistil. Fertilization of flowers is a key step in the plant life cycle. Successful pollination activates a series of events followed by fertilization and embryogenesis. This includes the development of an ovary on one hand, together with senescence and abscission of floral organs, degradation of macromolecules, and recycling of different nutrients on the other hand (O’Neill, 1997; Tripathi and Tuteja, 2007; Borghi and Fernie, 2020). *Cannabis* is a dioecious plant, harboring either female or male reproductive organs. It is also a wind-pollinated plant, in which the pollination of flowers is not dependent on specific animal pollinators. Phytocannabinoids are most abundant in the female flower inflorescences (Flores-Sanchez and Verpoorte, 2008). Fertilized flowers, harboring seeds, are considerably less potent. Hence the term “sinsemilla,” Spanish for “without seed,” that defines plants associated with high psychoactive effects (Potter et al., 2018). In addition, it is a common work practice by *Cannabis* growers to eliminate male plants growing in a field to maintain the unfertilized inflorescences and maximize the phytocannabinoid concentrations. Therefore, it is likely that the content of secondary metabolites such as phytocannabinoids and terpenoids changes following the pollination and fertilization of *Cannabis* inflorescences. However, although mentioned in a few studies (Meier and Mediavilla, 1998; Potter, 2009; Russo and Marcu, 2017), this phenomenon was not studied in depth. In the last few years, an increasing number of *Cannabis* growers are moving from using cuttings from female “mother plants” to seeds. Even though the seeds are usually feminized, around 5–10% will be males, and thus the question about the effect of pollination on the phytocannabinoids and terpenoids expression becomes critical.

In order to gain more insight into the *Cannabis* metabolite regulation pathway, this work studied the effect of flower fertilization on the plant’s secondary metabolite accumulation. We used indoor growing methods together with analytical procedures in order to investigate the effect of fertilization on metabolite composition and concentration in *Cannabis* inflorescences, and specify which metabolites are affected and to what extent.

## Materials and Methods

### Chemicals and Reagents

Liquid chromatography-mass spectrometric (LC/MS) grade acetonitrile (catalog number 1.00029), methanol (1.06035), and water (1.15333); and gas chromatography (GC) headspace grade dimethyl sulfoxide (DMSO) (1.01900) were purchased from Mercury Scientific and Industrial Products Ltd. (Rosh Haayin, Israel). Ethanol, (catalog number 052541), acetic acid (010778) and n-Hexane (091484) were obtained from BioLab Ltd. (Jerusalem, Israel). Phytocannabinoid analytical standards (>98%) CBG, THC, CBD, CBC, CBN, Cannabigerolic acid (CBGA), Tetrahydrocannabinolic acid (THCA), Cannabidiolic acid (CBDA), Cannabinolic acid (CBNA), Cannabichromenic Acid (CBCA), (-)-Δ8-trans-tetrahydrocannabinol (Δ^8^-THC), tetrahydrocannabivarin (THCV), Cannabidivarin (CBDV), Cannabidivarinic acid (CBDVA) and Cannabicyclol (CBL) were purchased from Sigma-Aldrich (Rehovot, Israel); Cannabichromevarin (CBCV) was purchased from Cayman Chemical (Ann Arbor, MI, United States). Terpenoid analytical standards (>95% unless stated otherwise) were purchased from Sigma-Aldrich (Rehovot, Israel); valencene (>80% pure), α- and β-curcumene (>90% pure), α-phellandrene, and sabinene were purchased from Extrasynthese (Genay, France); a mixture of n-alkanes was purchased from Sigma R 769 (40 mg/mL, C8-C20, Saint Louis, MO, United States) for semi-quantitative analysis.

### Experimental Design

The effect of fertilization was tested on two *Cannabis sativa* L. female plants. Female strains 333 THC-rich (15% THCA, 0.07% CBDA) and 423 CBD-rich (0.33% THCA, 9% CBDA) were subjected to fertilization and two male plants strains, 319 THC-rich (progeny of high THC landrace Highland Thai, Seedsman seeds) and 405 CBD-rich (progeny of Cherry CBD), were used as pollen donors. In addition, the female plants were subjected to a sex conversion treatment (Mohan Ram and Sett, 1982; Small and Naraine, 2016) and these induced males were also used as pollen donors to fertilize the two female plants. In order to achieve pollen sacs, 45 days old rooted cutting, 30 cm size female plants were sprayed daily until completely moist with ethylene inhibitor (Sodium Thiosulfate 0.5%) for 5 days prior to transferring to short day conditions. The female plants that were sex changed are referred to as males or induced-males. The female *Cannabis* plants were grown, three plants for each treatment, under an 18/6 light/dark regime (800 μmol), 23–27°C for 30 days before being transferred to flowering chambers with a 12/12 light/dark regime (500 μmol), 23–27°C for up to either 42 (6 weeks) or 56 (8 weeks) days before some inflorescences were removed for chemical analysis. Female plants were grown in small flowering chambers (1 m^2^) in the presence of a single pollen donor. All plants were grown in 1 L pots on a mixture of pit/coconut 70%/30% soil, respectively.

### Extraction and Sample Preparation of Phytocannabinoids

The inflorescences of the treated plants, 3–4 apical inflorescences per plant, were harvested and dried for 24–48 h at 40°C until they reached a moisture content of 12% weight for weight (w/w). The inflorescences were ground to a fine powder using an electric grinder, then 98–103 mg were weighed and extracted with 1 mL ethanol. Samples were sonicated in an ultrasonic bath for 30 min, agitated in an orbital shaker at 25°C for 20 min, centrifuged at 20,000 x g for 5 min, then the samples were dissolved and diluted x20 in ethanol and filtered through a 0.22 μm Polytetrafluoroethylene syringe filter (Lumitron Ltd., Petah Tikva, Israel) prior to analysis.

### Phytocannabinoid Identification and Quantification

Phytocannabinoid analyses for high concentrations of THC and CBD were performed using a Thermo Scientific UltiMate 3000 ultra-high-performance liquid chromatography coupled with an ultraviolet-visible diode array detector (UHPLC/UV) system. All other phytocannabinoids were identified and quantified by a similar UHPLC instrument coupled with a Q Exactive^TM^ Focus Hybrid Quadrupole-Orbitrap MS (Thermo Scientific, Bremen, Germany), as previously described (Berman et al., 2018; Milay et al., 2020). In short, chromatographic separation was achieved using a HALO C18 Fused-Core column (2.7 μm, 150 × 2.1 mm), with a HALO guard column (2.7 μm, 5 × 2.1 mm), and a ternary A/B/C multistep gradient (solvent A: water with 0.1% acetic acid, solvent B: acetonitrile with 0.1% acetic acid, and solvent C: methanol). Identification and absolute quantification of phytocannabinoids were performed by external calibrations, as previously described (Berman et al., 2018). Sixteen analytical standards (CBDVA, CBDA, CBCA, CBNA, CBGA, THCA, CBDV, CBD, CBC, CBN, CBG, THC, Δ8-THC, CBL, THCV, CBCV) were used for direct quantification and semi-quantification of additional phytocannabinoids. All extracted samples were injected and analyzed by electrospray ionization (ESI)-LC/MS analysis, diluted at ratios of 1:9, 1:99, and 1:999 v/v *Cannabis* extract to ethanol.

### Terpenoids Identification and Quantification

Profiling of terpenoids was performed using a modification of the static headspace gas chromatography tandem MS (SHS-GC/MS/MS) method by full evaporation technique (Shapira et al., 2019). SHS-injections were performed by PAL RTC robotic tool (CTC Analytics, Swaziland) with 30 min incubation time, temperature of 140°C and 1,000 μL injection volume of the gas phase. Gas chromatographic separation was achieved in 74 min using a TRACE 1310 GC (Thermo Fisher Scientific, Bremen, Germany) equipped with a 30 m × 0.25 mm × 0.25 μm capillary DB-35MS UI column (Agilent Technologies, United States). MS/MS compounds detection was performed by a TSQ 8000 Evo triple quadrupole mass spectrometer (Thermo Fisher Scientific, Bremen, Germany). For the terpenoids analyses, 10 mg of each ground *Cannabis* sample was weighed in duplicates in a 20 mL HS amber vial with 1.2 g of glycerol and sealed by a magnetic cap. Solutions for the construction of the calibration curves were prepared in hexane and then 10 μL for each calibration level was added to amber vials with 1.2 g of glycerol in the same manner as the samples.

Some of the terpenoids were calculated semi-quantitatively based on the calibration curves of terpenoids with commercially available analytical standards with similar MS spectral characteristics and retention times. Identification of these terpenoids was performed by spectral searching against the NIST library (version 2.2) and relative Kovats retention indexes using a mixture of n-alkanes run under the same chromatographic conditions (for full details see Supplementary Tables 2, 3).

### Statistical Analysis

Statistical analyses were conducted using GraphPad Prism software version 8.2.1 (GraphPad Inc.). Differences between samples in phytocannabinoid and terpenoid concentrations were analyzed using two-way ANOVA followed by Dunnett’s multiple comparison test. *P*-values were corrected for multiple testing using the Tukey *post hoc* test. A value of at least *p* ≤ 0.05 was considered significant for all tests (*p ≤ 0.05, **p ≤ 0.01, ***p ≤ 0.001, ^****^p ≤ 0.0001). Outliers were defined as data points greater than two standard deviations from the mean (9.6 for THCA and 9.5 for CBDA).

## Results

### Phytocannabinoids Quantity Predominantly Decreases After Fertilization

**FIGURE 1:**
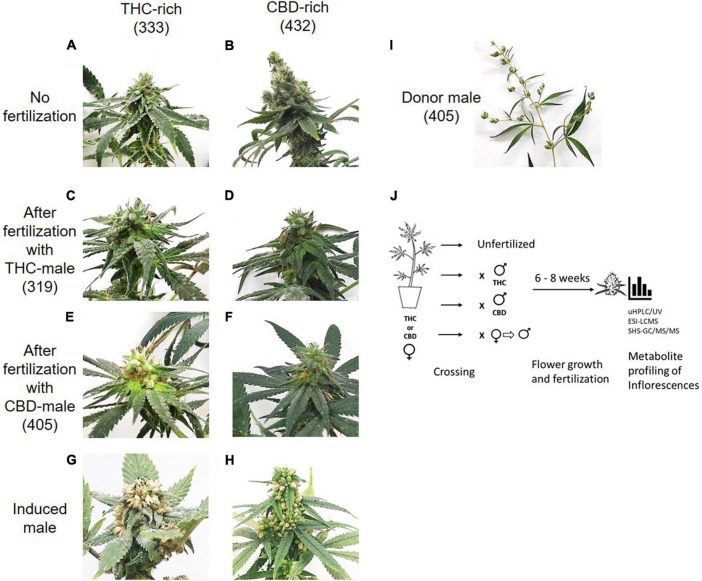
Study design. Differences in inflorescences between THC-rich and CBD-rich plants without **(A,B)** and after fertilization with either THC-rich male (strain 319) **(C,D)** or CBD-rich male (strain 405) **(E,F)** and their respective induced-male plants **(G,H)**. **(I)** Representative image of a male donor plant (strain 405). To capture images, the plants were placed on the same white background and photographed individually. **(J)** Experimental design. Female *Cannabis* plants of two distinct types, THC- or CBD-rich chemovars, were subjected to fertilization by three different male *Cannabis* plants: THC- or CBD-rich plants, and an induced-male plant achieved by application of ethylene inhibitor. Female and male plants were incubated together for 6–8 weeks. The profile of their secondary metabolites was analyzed by UHPLC/UV and ESI-LC/MS for phytocannabinoids and SHS-GC/MS/MS for terpenoids.

Mature inflorescences (6 or 8 weeks post flower induction) from female *Cannabis* plants of two distinct chemovars (Figures 1A,B), THC-rich (Type I) and CBD-rich (Type III), were subjected to fertilization by three different male *Cannabis* plants: THC-rich (Figures 1C,D), CBD-rich (Figures 1E,F) or the original female plant induced to develop male pollen sacs by application of ethylene inhibitor (Figure 1). Induced-male plants (Figures 1G,H) were genetically identical to the female plants, had a distinct change in the sex of the flowers after treatment and a larger number of inflorescences compared to males (Figure 1I). Specific fertilization was achieved by incubation of the individual plants (Figure 1J).

**FIGURE 2:**
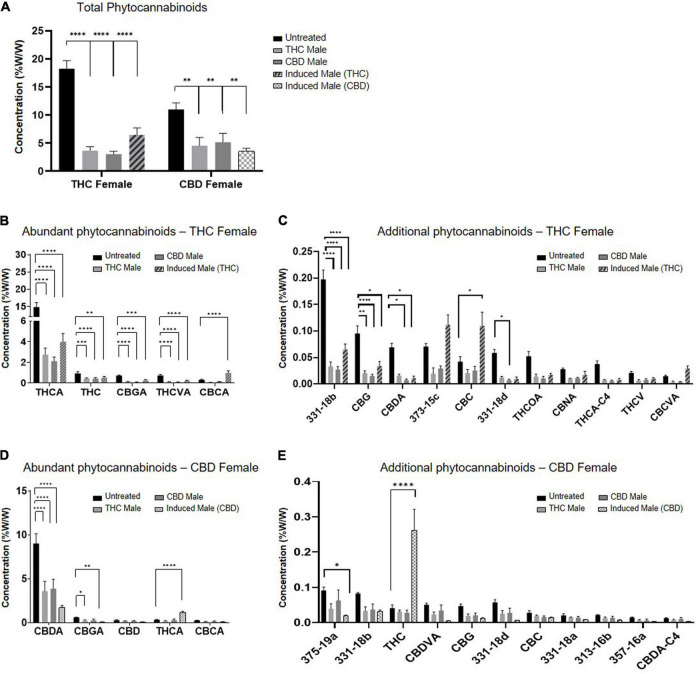
Phytocannabinoids quantity predominantly decreases after fertilization with all types of males. **(A)** Total phytocannabinoid concentrations and **(B–E)** Individual phytocannabinoid concentrations after fertilization relative to unfertilized control. Abundant phytocannabinoid concentrations were considered > 0.2% **(B,D)** and additional phytocannabinoid concentrations were 0.001–0.2% **(C,E)** in the unfertilized plants. Data are presented as mean ± SEM (*n* = 4–6, %w/w) and statistically analyzed by two-way ANOVA followed by Dunnett’s multiple comparison test (*p ≤ 0.05; **p ≤ 0.01; ***p ≤ 0.001; ****p ≤ 0.0001). Significance in C, E was calculated after excluding THCA and CBDA, respectively, from the data.

Fertilization resulted in a predominantly significant decrease of overall total phytocannabinoids concentration in inflorescences for both the THC-rich and CBD-rich females, by all three types of males (Figure 2A). The concentration of the phytocannabinoids was analyzed by UHPLC/UV and electrospray ionization-liquid chromatography/mass spectrometry (ESI-LC/MS). The full list of the 95 phytocannabinoids quantified is displayed in Supplementary Table 1 (as named by Berman et al., 2018). A sharper decrease was detected in the THC-rich chemovar female, exhibiting an average 75% decrease, while CBD-rich females showed a 60% decrease in phytocannabinoid contents after fertilization. Next, we investigated changes in quantities of individual phytocannabinoids (Figures 2B–E). For the THC-female, fertilization caused a reduction in the abundant phytocannabinoids, whose concentrations in the plant were above 0.02%, except for the phytocannabinoid (CBCA), which had an increase of about 50% when the plant was fertilized with an induced male (Figure 2B). Additional phytocannabinoids, whose concentrations in the plant were 0.001–0.2%, were also mostly reduced upon fertilization. The concentrations of CBC, cannabichromevarinic acid (CBCVA) and 373-15c were increased when fertilized by the induced male (Figure 2C). When THCA was excluded as an outlier as its concentration is 15-fold higher, the less abundant phytocannabinoids 331-18b, CBG, CBDA and 331-18d were significantly reduced upon fertilization. Similarly, for the CBD-female, fertilization caused a reduction in both the abundant (Figure 2D) and additional phytocannabinoids when CBDA is excluded as an outlier (Figure 2E), except for the concentrations of THCA and THC that increased after fertilization with the induced male.

### Terpenoids Quantity Decreases After Fertilization in the Cannabidiol-Rich Female Plant but Varies in the THC-Rich Female Plant

**FIGURE 3:**
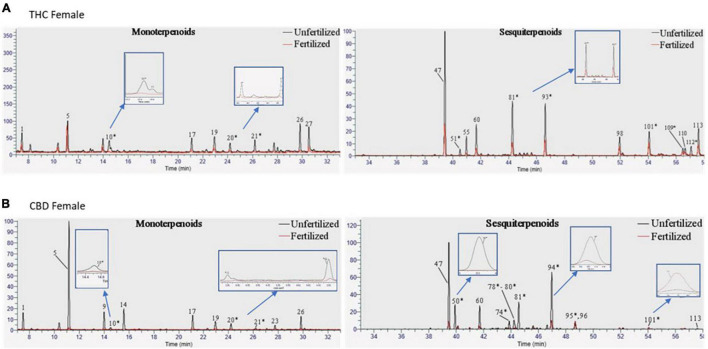
Terpenoid profiles of *Cannabis* strains before and after fertilization. Overlay of chromatograms of the unfertilized and fertilized samples of **(A)** THC-rich and **(B)** CBD-rich female plants were performed by the same scales [retention time (RT); relative abundance of the signal intensity; weight of the samples (10 mg)], showing monoterpenoids on the left and sesquiterpenoids on the right. * Terpenoids that were semi-quantified.

In addition to assessing the phytocannabinoid contents, we quantified over 100 terpenoid compounds. The THC- and CBD-rich female plants differed in their profile of terpenoids before fertilization (Supplementary Figure 1). About half of the quantified terpenoids had pure analytical standards available and were analyzed as previously described (Shapira et al., 2019). However, out of a total of 113 terpenoids detected using (SHS-GC/MS/MS), 63 terpenoids in either the THC-rich or the CBD-rich plants did not have commercially available standards (for a full list see Supplementary Table 4). Some of these terpenoids demonstrated significant changes after fertilization, therefore, we assessed them with a newly developed semi-quantitative analysis (Figure 3). In this manner, we quantified terpenoids such as δ-Guaiene and *trans*-α-Bisabolene (denoted as 81 and 93, respectively). The semi-quantitative analysis is based on the calibration curves of terpenoids with commercially available analytical standards, relying primarily on similar MS spectral characteristics and also on retention times (Supplementary Figure 2).

**FIGURE 4:**
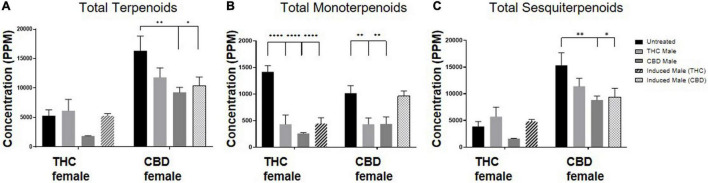
Terpenoid quantity varies after fertilization. Terpenoid concentrations as quantified by SHS-GC/MS/MS of **(A)** total identified terpenoids, **(B)** total monoterpenoids and **(C)** total sesquiterpenoids. Data are reported as mean ± SEM of terpenoid concentrations (*n* = 3–4, ppm). Statistically significant differences between treatments and control (unfertilized) were calculated by two-way ANOVA followed by Dunnett’s multiple comparison test (*p ≤ 0.05; **p ≤ 0.01; ****p ≤ 0.0001).

The total amount of terpenoids in the inflorescences was found to be chemovar specific (Figure 4A). The high-CBD female plants exhibited two to threefold higher concentrations of terpenoids, both in the unfertilized and all three types of fertilized plants, compared to the THC-rich female plants. Upon fertilization, there were no significant changes in terpenoid accumulation in the THC-rich female. In the CBD-rich female plants, there was no significant change when fertilized with a THC-rich male plant, but fertilization with a CBD-rich male or an induced male resulted in a significant reduction in total terpenoids. The profile of terpenoids in plants is highly variable (Booth et al., 2020), and being mostly volatile compounds, they are also more susceptible to changes due to sample preparation procedure, e.g., the freshness of samples (Livingston et al., 2020). We detected an overall fertilization-dependent decrease in total terpenoid accumulation only in the CBD-rich plant, while the THC-rich plant showed a mixed trend of changes, either reduction or no significant change.

Out of 113 terpenoids detected, 31 were monoterpenoids, built up by two isoprene units (10 carbons) and the rest were sesquiterpenoids, built up by three isoprene units (15 carbons) (Shapira et al., 2019). To further evaluate the influence of fertilization on terpenoid accumulation after fertilization, we analyzed these two distinct subgroups. Monoterpenoid concentrations were significantly reduced for the THC-rich female by 60–80% upon fertilization with all three types of males; for the CBD-rich female, there was a significant 50% reduction except for when fertilized by the induced-male, which left the concentrations unchanged (Figure 4B). The concentration of sesquiterpenoids was unchanged for the THC-rich female, but there was a trend of reduced concentrations in the CBD-rich fertilized female, which was statistically significant when fertilized with the CBD-rich or the induced male (Figure 4C).

### Individual Terpenoid Concentrations Are Differentially Affected by Fertilization

**FIGURE 5:**
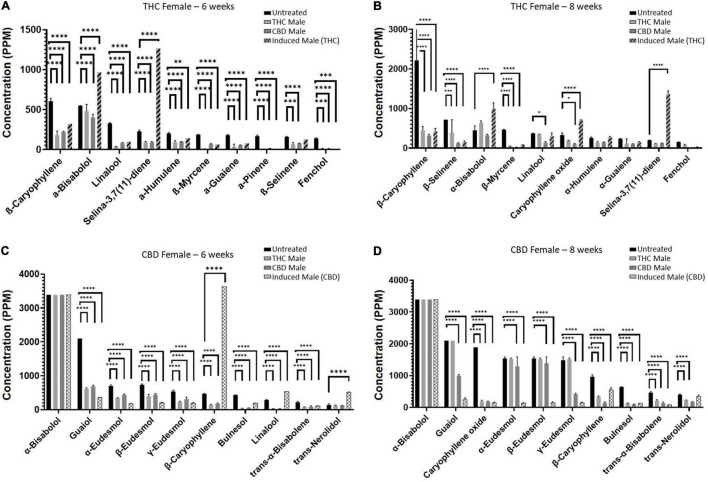
Individual terpenoid concentrations in THC- and CBD-rich female plants at 6 or 8 weeks. Abundant terpenoids in the unfertilized female flowers and their concentrations at 6 weeks **(A,C)** and 8 weeks **(B,D)**. Data are reported as mean ± SEM of terpenoid concentration (*n* = 2–3, except THC-female fertilized by induced male (THC) at 6 weeks). Statistically significant differences between treatments and control (unfertilized) were calculated by two-way ANOVA followed by Dunnett’s multiple comparison test (*p ≤ 0.05; **p ≤ 0.01; ***p ≤ 0.001; ****p ≤ 0.0001). Values presented without SEM exceeded the maximal detection limit (maximum limits of detection for terpenoids appear in Supplementary Table 5).

Next, we set out to examine the accumulation of individual terpenoids in the plants (Figure 5) and found chemovar-specific differences. For the THC-rich female, the most abundant terpenoid was β-caryophyllene and its concentration was reduced upon fertilization (Figures 5A,B). For the CBD-rich female, the most abundant terpenoid was α-bisabolol, its concentration was above detection limit both before and after fertilization (Figures 5C,D). Moreover, we noticed that the terpenoid profile changed during the length of the flowering time, between 6–8 weeks after fertilization. This was in contrast to the phytocannabinoids profile, which was more consistent between these two time-points (data not shown). For example, for the CBD-rich female, the sesquiterpenoid Caryophyllene oxide had a very low concentration in the 6-week flowering plant but became highly abundant in the 8-week plant (Figures 5C,D). Hence, in addition to chemovar-specific differences, differential accumulation was observed between 6- and 8-week growth in the same chemovar.

**FIGURE 6:**
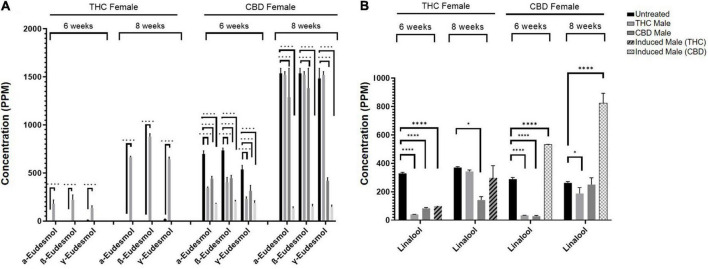
Specific terpenoids are increased following fertilization. **(A)** α-, β-, and γ-Eudesmol and **(B)** Linalool concentrations in THC-rich and CBD-rich females at 6-weeks and 8-weeks after fertilization (*n* = 2–3, except THC-female fertilized by induced male (THC) at 6 weeks). Statistically significant differences between treatments and control (unfertilized) were calculated by two-way ANOVA followed by Dunnett’s multiple comparison test (*p ≤ 0.05; ****p ≤ 0.0001).

As seen in Figure 5, numerous terpenoids significantly decreased following fertilization. However, several specific terpenoids showed an interesting increase in concentration after fertilization. For example, in the THC-rich female, members of the Eudesmol family of sesquiterpenoids (α-, β-, and γ-Eudesmol) were mostly undetected in the unfertilized plant, but their concentrations were significantly increased upon fertilization by the THC male plant only, both at 6 and 8 weeks after fertilization (Figure 6A). Interestingly, the levels of these terpenoids were either reduced or unchanged in the CBD-rich female due to fertilization processes. In contrast, in the CBD-rich female, the monoterpenoid Linalool significantly increased upon fertilization by the induced male, but was reduced or unchanged following all other fertilization processes in both plant chemovars (Figure 6B).

## Discussion

The present study was designed to examine the influence of flower fertilization on the accumulation of *Cannabis* secondary metabolites. The primary outcome is the significant overall decrease in phytocannabinoid metabolites upon fertilization. This decrease was evident in almost all phytocannabinoids measured, regardless if those were the abundant ones or the relatively low accumulating components (Figure 2). Though the altogether amount of phytocannabinoids is drastically reduced, the ratio between the different compounds is kept and their profile in the plant remains principally unchanged.

Terpenoid concentrations mostly decreased but varied. While monoterpenoids had a similar decrease as portrayed by the phytocannabinoids, sesquiterpenoids exhibited a more diverse pattern, some of which increased and some decreased upon fertilization (Figure 3). However, examining specific metabolites can point to several phytocannabinoids or terpenoids that have an individual trend, suggesting a more complex regulatory network (Figures 4, 5).

First, these results confirm that when the objective is to maintain high levels of phytocannabinoids, fertilization must be avoided. Apart from a physical separation between female and male flowers or vegetative reproduction, this goal could be achieved using advanced genetic manipulations that target female fertilization pathways (Huang et al., 2016; Jung et al., 2020; Zhu et al., 2020).

**FIGURE 7:**
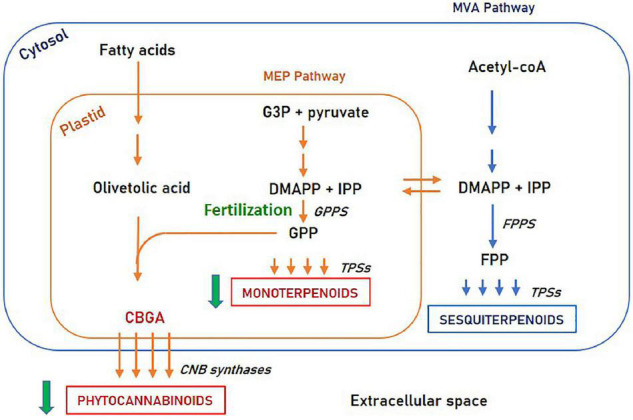
Phytocannabinoids and terpenoids biosynthesis pathways. Fertilization affects the MEP pathway in an enzymatic step upstream to GPP synthase. MEP, Methylerythritol phosphate; MVA, Mevalonate; G3P, Glyceraldehyde 3-phosphate; DMAPP, Dimethylallyl diphosphate; IPP, Isopentenyl diphosphate; GPP, Geranyl diphosphate; FPP, Farnesyl diphosphate; GPPs, GPP synthase; FPPS, FPP synthase; TPSs, Terpene synthases.

Second, this study revealed the resemblance between monoterpenoids and phytocannabinoids accumulation patterns. Both secondary metabolite species are decreased upon fertilization, while sesquiterpenoids are differently influenced. Possible explanations for this similarity are common intracellular regulation pathways or shared morphological structures. From a cellular perspective, monoterpenoids and phytocannabinoids share the common biosynthetic precursor Geranyl diphosphate (GPP) and are both biosynthesized in the plastid (Booth et al., 2020; Romero et al., 2020). In contrast, sesquiterpenoids are synthesized in the cytosol from a different precursor (Farnesyl pyrophosphate—FPP). This suggests that phytocannabinoids and monoterpenoids may share a common regulation mechanism, involving an enzymatic step upstream to GPP, such as GPP synthase (illustrated in Figure 7).

Alternatively, from a morphological perspective, previous studies have shown that although phytocannabinoids, monoterpenoids, and sesquiterpenoids are all biosynthesized and accumulated in the glandular trichomes, their distribution differentiates during trichome development and between trichome types. A recent study by Booth et al. (2020) showed an increase in the ratio of monoterpenoids relative to sesquiterpenoids when flowers are maturing. Another study (Livingston et al., 2020) showed that monoterpenoids are accumulated in both pre-stalked and stalked trichomes, while sesquiterpenoids are abundant in sessile trichomes. Phytocannabinoids are accumulated in both types of trichomes, but the stalked type composed 80–90% of the total trichomes in the mature flower. A common accumulation pattern of monoterpenoids and phytocannabinoids during flower development was also previously demonstrated (Aizpurua-Olaizola et al., 2016). Parallel accumulation and decrease of phytocannabinoids and monoterpenoids in contrast to sesquiterpenoids may suggest that trichome types are differently affected by fertilization, and hence the diversity in metabolite accumulation.

An additional major finding depicted in this study is the somewhat dependent outcome of the fertilization process on the pollen donor plant. Both THC- or CBD-rich male plants, whether naturally occurring or female-induced, had a different impact on the metabolite concentration in the female after fertilization. For instance, fertilization by the induced-male led to an increase of specific phytocannabinoids (Figure 2): THC and THCA in the CBD female and CBC, CBCA, CBCVA, and 373-15C in the THC female. The exact mechanism by which these phytocannabinoids are increased is not yet clear. It may be the result of altered regulation of synthesis enzymes, for example the upregulation of THCA synthase or CBCA synthase (Laverty et al., 2019). A previous study found over 10,000 genes are differentially expressed upon masculinization of female plants (Adal et al., 2021), but it is not clear how these genes are related to phytocannabinoid expression in the fertilized female plant. A donor-dependent effect was also detected in the specific increase in the Eudesmol family (α-, β-, and γ-Eudesmol) components, which were highly increased in the THC-rich female upon fertilization by the THC-rich male plant (Figure 6A) and a parallel specific increase in Linalool in the CBD-rich female fertilized by the induced male (Figure 6B). However, regardless of the type of male plant used for fertilization, the overall profile of the phytocannabinoids in the fertilized female plant remained unaltered, i.e., no new phytocannabinoids that were not expressed in the unfertilized plant were discovered and the relative ratio between the different phytocannabinoids was mostly kept. Interestingly, though the density of phytocannabinoids and terpenoids in males is minor (data not shown) compared to the female flowers, with high potency female plants showing 10–20-times more THC than corresponding males (Clarke and Merlin, 2016), male plants also possess a distinct profile of these compounds.

## Conclusion

Here, we used highly advanced analytical methods to thoroughly assess the composition of 95 phytocannabinoids and 113 terpenoids in the inflorescences of female plants fertilized by different males, including the female plant itself induced to develop male pollen sacs. We found that fertilization significantly decreased phytocannabinoids content, while terpenoids were differentially affected. To further elucidate the effect of fertilization on the secondary metabolite accumulation, future studies that follow the gene expression of enzymes upstream to GPP after fertilization may allow exposing master regulators of the biochemical pathways. In addition, better characterization of the morphological changes following fertilization may shed light on how different trichome types are affected by fertilization. Finally, the variance in metabolites observed by fertilization with different male plants may suggest that the pollen itself or the developing embryo influence the female sporophyte.

Altogether, one must remember that these specialized secondary metabolites have an important role *in planta*, increasing the plant fitness to the environment (Huchelmann et al., 2017). The substantial decrease in phytocannabinoids and terpenoids after fertilization may point to their functional roles in the plant. The actual functions of phytocannabinoids and terpenoids in *Cannabis* were only sparsely studied, mainly suggesting roles in defense against biotic or abiotic factors (Potter, 2009), protection from UV radiation (Eichhorn Bilodeau et al., 2019), prevention of desiccation (Gülck and Møller, 2020), or induction of cell death in leaves (Morimoto et al., 2007). The observed dynamics of the accumulation of these metabolites during flower development and fertilization may point to their different roles along the plant’s life cycle.

## Data Availability Statement

The raw data supporting the conclusions of this article will be made available by the authors, without undue reservation.

## Author Contributions

PB, MF, and DM: conception and design. OC, IK, and RP: plant maintenance, manipulations, fertilization, and harvest. CL: acquisition and ESI-LC/MS analysis and interpretation of data. AS: acquisition of GC data and development of semi-quantitative GC methodology. OG: extraction and sample preparation of Cannabis samples and UHPLC/UV analysis. CL, AS, and SP: figure preparation. CL, SP, PB, MF, and DM: writing, review, and revision of the manuscript. MF and DM: study supervision. All authors contributed to the article and approved the submitted version.

## Conflict of Interest

The authors declare that the research was conducted in the absence of any commercial or financial relationships that could be construed as a potential conflict of interest.

## Publisher’s Note

All claims expressed in this article are solely those of the authors and do not necessarily represent those of their affiliated organizations, or those of the publisher, the editors and the reviewers. Any product that may be evaluated in this article, or claim that may be made by its manufacturer, is not guaranteed or endorsed by the publisher.
