# Supplementary material for: Fertilization Following Pollination Predominantly Decreases Phytocannabinoids Accumulation and Alters the Accumulation of Terpenoids in Cannabis Inflorescences
Source: Front Plant Sci. 2021 Nov 5;12:753847. doi: 10.3389/fpls.2021.753847 (PMC8602813; doi:10.3389/fpls.2021.753847)
Supplement: Supplementary file 1 [file Data_Sheet_1.pdf]

**Supplemental Materials**

**Supplemental Tables**

Table S1. List of full and abbreviated names of phytocannabinoids

Table S2. Calibration curves used for quantification of terpenoids without commercially available standards

Table S3. SRM fragmentation of the terpenoids without commercially available standards

Table S4. List of identified terpenoids without commercially available standards

Table S5. List of terpenoids maximal detection concentration

**Supplemental Figures**

Figure S1. Terpenoid profiles of CBD- and THC-rich unfertilized strains of Cannabis

Figure S2. Principles of terpenoid semi-quantification

**Table S1. List of full and abbreviated names of phytocannabinoids**

| **1. Cannabigerol (CBG) type** | **Abbreviated name** | **Acid Form** |
|---|---|---|
| Cannabigerol-C5 | CBG | CBGA |
| Cannabigerobutol-C4 | CBG-C4 / CBGB | CBGA-C4 |
| Cannabigerovarin-C3 | CBGV | CBGVA |
| Cannabigerorcol-C5 | CBGO | CBGOA |
| Sesquicannabigerol-C5 | SesquiCBG | Sesqui-CBGA |
| Cannabigerol monomethyl ether-C5 | CBGM | CBGMA |
| **2. Δ$^{9}$-*trans*-tetrahydrocannabinol (Δ$^{9}$-THC) type** | | |
| (-)-Δ$^{9}$-*trans*-Tetrahydrocannabinol-C5 | THC | THCA |
| (-)-Δ$^{9}$-*trans*-tetrahydrocannabutol-C4 | THC-C4 / THCB | THCA-C4 |
| (-)-Δ$^{9}$-*trans*-Tetrahydrocannabivarin-C3 | THCV | THCVA |
| (-)-Δ$^{9}$-*trans*-Tetrahydrocannabiorcol-C1 | THCO | THCOA |
| (-)-Δ9-trans-Tetrahydrocannabinol monomethyl ether-C5 | THCM | THCMA |
| **3. Cannabidiol (CBD) type** | | |
| (-)-Cannabidiol-C5 | CBD | CBDA |
| (-)-Cannabidibutol-C4 | CBD-C4 / CBDB | CBDA-C4 |
| (-)-Cannabidivarin-C3 | CBDV | CBDVA |
| (-)-Cannabidiorcol-C1 | CBDO | CBDOA |
| Cannabidiol monomethyl ether-C5 | CBDM | CBDMA |
| **4. Cannabichromene (CBC) type** | | |
| (±)-Cannabichromene-C5 | CBC | CBCA |
| (±)-Cannabibutol-C4 | CBC-C4 / CBCB | CBCA-C4 |
| (±)-Cannabichromevarin-C3 | CBCV | CBCVA |
| (±)-Cannabiorchromorcol-C1 | CBCO | CBCOA |
| **5. Cannabinol (CBN) type** | | |
| Cannabinol-C5 | CBN | CBNA |
| Cannabibutol-C4 | CBN-C4 / CBNB | CBNA-C4 |
| Cannabivarin-C3 | CBNV | CBNVA |
| Cannabiorcol-C1 | CBNO | CBNOA |
| Cannabinodivarin-C3 | CBNDV | |
| 8-hydroxycannabinol | CBN-8-OH | CBNA-8-OH |
| Cannabinol monomethyl ether-C5 | CBNM | |
| **6. Δ$^{8}$-trans-tetrahydrocannabinol (Δ$^{8}$-THC) type** | | |
| (-)-Δ$^{8}$-*trans*-(6aR,10aR)-Tetrahydrocannabinol-C5 | Δ$^{8}$-THC | |
| **7. Cannabicyclol (CBL) type** | | |
| (±)-(1aS,3aR,8bR,8cR)-Cannabicyclol-C5 | CBL | |
| **8. Cannabinodiol (CBND) type** | | |
| Cannabinodiol-C5 | CBND | CBNDA |
| **9. Cannabielsoin (CBE) type** | | |
| (5aS,6S,9R,9aR)-Cannabielsoin-C5 | CBE | CBEA |
| Cannabielsoin-C3 | CBEV | CBEVA |
| **10. Cannabitriol (CBT) type** | | |
| (±)-*cis/trans*-Cannabitriol-C5-1 | CBT-1 | CBTA-1 |
| (±)-*cis/trans*-Cannabitriol-C5-2 | CBT-2 | |
| (±)-*cis/trans*-Cannabitriol-C5-3 | CBT-3 | CBTA-3 |
| Cannabitriol-C3-1 | CBTV-1 | |
| Cannabitriol-C3-3 | CBTV-3 | |

**11. Phytocannabinoids without absolute identification. Names prescribed by (Berman et al.,2018)**

| | |
|---|---|
| 313-16b | 361-17a |
| 327-13a | 361-17b |
| 327-13b | 371-14a |
| 327-13c | 371-14b |
| 329-11a | 373-12a |
| 329-11b | 373-12b |
| 329-11c | 373-12c |
| 329-11d | 373-12d |
| 329-11e | 373-15b |
| 331-18a | 373-15c |
| 331-18b | 375-19a |
| 331-18c | 375-19b |
| 331-18d | 375-19c |
| 357-16a | 417-15a |

**Table S2. Calibration curves used for quantification of terpenoids without commercially available standards**

| Terpenoid without STD | STDcurve |
|---|---|
| β-Phellandrene | α-Phellandrene |
| $C_{10}H_{18}O$-154 (93/79/99/121)-1 | Linalool |
| $C_{10}H_{18}O$-154 (93/79/99/121)-2 | Linalool |
| α-Cubebene | Isoledene |
| Ylangene | Isoledene |
| α-Copaene | α-Cedrene |
| 7-epi-Sesquithujene | α-Cedrene |
| $C_{15}H_{24}$-204 (105/(120+119)/161) | Isoledene |
| β-Cubebene | Isoledene |
| Sesquithujene | *trans*-β-Caryophyllene |
| β-Isocomene | Sativene |
| α-Santalene | Cyclosativene |
| cis-α-Bergamotene | α-Cedrene |
| trans-α-Bergamotene | α-Cedrene |
| α-Guaiene | Aromadendrene |
| γ-Elemene | α-Cedrene |
| β-Santalene | α-Humulene |
| Guaia-6,9-diene | Aromadendrene |
| $C_{15}H_{24}$-204 (69/91/105/161) | *trans*-β-Farnesene |
| $C_{15}H_{24}$-204 (91/105/161) | Aromadendrene |
| $C_{15}H_{24}$-204 (161/105/133/91) | Valencene |
| $C_{15}H_{24}$-204 (105/91/133/161/189) | Aromadendrene |
| Acoradiene | Aromadendrene |
| $C_{15}H_{24}$-204 (105)-1 | Aromadendrene |
| γ-Curcumene | β-Curcumene |
| $C_{15}H_{24}$-204 (133/189)-1 | β-Chamigrene |
| Sesquisabinene | Ledene |
| γ-Muurelene | Valencene |
| α-Amorphene | Ledene |
| Aristolochene | Ledene |
| Germacrene D | Ledene |
| $C_{15}H_{24}$-204 (133/189)-2 | β-Chamigrene |
| $C_{15}H_{24}$-204 (119/93/161) | β-Curcumene |
| α-Selinene | Ledene |
| β-Selinene | β-Chamigrene |
| α-Farnesene | *trans*-β-Farnesene |
| β-Bisabolene | *trans*-β-Farnesene |
| δ-Guaiene | Ledene |
| β-Cadinene- $C_{15}H_{24}$-204(119/161/105/134) | β-Curcumene |
| Dihydroagarofuran | β-Curcumene |

| | |
|---|---|
| $C_{15}H_{24}$-204 (similar to Germarcene B) | β-Curcumene |
| Sesquicineole | β-Curcumene |
| Eremophilene | Valencene |
| β-Sesquiphellandrene | *trans*-β-Farnesene |
| γ-Cadinene | Valencene |
| δ-Cadinene | β-Curcumene |
| $C_{15}H_{24}$-204 (105)-2 | Ledene |
| α-Panasinsene | β-Curcumene |
| *trans*-α-Bisabolene | α-Humulene |
| Selina-3,7(11)-diene | Valencene |
| Germacrene B | β-Curcumene |
| Guaia-3,9-diene | β-Curcumene |
| α-epi-7-epi-5-Eudesmol | Globulol |
| $C_{15}H_{26}O$-222 (similar to γ-Eudesmol) | β-Eudesmol |
| Humulene oxide II | Caryophyllene oxide |
| Selin-6-en-α-ol | β-Eudesmol |
| Hinesol | α-Bisabolol |
| γ-Eudesmol | β-Eudesmol |
| $C_{15}H_{26}O$-222 (105/161/59)-1 | Globulol |
| Agarospirol | α-Bisabolol |
| $C_{15}H_{26}O$-222 (105/161/59)-2 | Globulol |
| $C_{15}H_{26}O$-222 (59/81/107 /149/161) | β-Eudesmol |
| α-Eudesmol | β-Eudesmol |
| 7-epi-α-Eudesmol | α-Bisabolol |
| Bulnesol | α-Bisabolol |

**Table S3. SRM fragmentation of the terpenoids without commercially available standards**

| Compound name | RT [min] | Precursor I [m/z] | Product I [m/z] | CE [eV] | Ratio |
|---|---|---|---|---|---|
| β-Phellandrene | **14.50** | **93** | **77** | **10** | **Quan** |
| | | 93 | 91 | 6 | 75 |
| | | 77 | 51 | 14 | 32 |
| $C_{10}H_{18}O$-154 (93/79/99/121)-1 | **24.10** | **93** | **77** | **12** | **Quan** |
| | | 99 | 43 | 8 | 42 |
| | | 121 | 93 | 8 | 23 |
| $C_{10}H_{18}O$-154 (93/79/99/121)-2 | **26.20** | **93** | **77** | **12** | **Quan** |
| | | 99 | 43 | 8 | 40 |
| | | 121 | 93 | 8 | 25 |
| α-Cubebene | **34.61** | **105** | **77** | **18** | **Quan** |
| | | 119 | 91 | 10 | 80 |
| | | 161 | 105 | 8 | 76 |
| Ylangene | **36.06** | **105** | **77** | **16** | **Quan** |
| | | 119 | 91 | 12 | 96 |
| | | 161 | 105 | 6 | 59 |
| α-Copaene | **36.22** | **119** | **91** | **12** | **Quan** |
| | | 105 | 77 | 20 | 80 |
| | | 91 | 65 | 14 | 44 |
| | | 161 | 105 | 8 | 85 |
| 7-epi-Sesquithujene | **36.66** | **93** | **77** | **10** | **Quan** |
| | | 69 | 41 | 6 | 32 |
| | | 119 | 91 | 14 | 55 |
| $C_{15}H_{24}$-204 (105/(120+119)/161) | **36.84** | **120** | **105** | **8** | **Quan** |
| | | 119 | 91 | 12 | 30 |
| | | 105 | 77 | 18 | 44 |
| | | 161 | 105 | 8 | 18 |
| β-Cubebene | **37.68** | **105** | **77** | **16** | **Quan** |
| | | 105 | 79 | 12 | 70 |
| | | 119 | 91 | 12 | 180 |
| | | 161 | 105 | 10 | 80 |
| Sesquithujene | **37.70** | **93** | **77** | **10** | **Quan** |
| | | 69 | 41 | 6 | 35 |
| | | 91 | 65 | 16 | 30 |
| | | 119 | 91 | 14 | 55 |
| β-Isocomene | **37.80** | **108** | **93** | **5** | **Quan** |
| | | 107 | 91 | 10 | 32 |
| | | 93 | 77 | 12 | 64 |

| | | | | | |
|---|---|---|---|---|---|
| *cis*-α-Bergamotene | **38.25** | **119** | **91** | **14** | **Quan** |
| | | 69 | 41 | 6 | 70 |
| | | 93 | 91 | 6 | 140 |
| | | 107 | 91 | 10 | 60 |
| α-Santalene | **38.34** | **94** | **79** | **10** | **Quan** |
| | | 41 | 39 | 6 | 20 |
| | | 95 | 67 | 10 | 18 |
| *trans*-α-Bergamotene | **39.01** | **119** | **91** | **8** | **Quan** |
| | | 69 | 41 | 6 | 85 |
| | | 107 | 91 | 10 | 23 |
| α-Guaiene | **39.67** | **105** | **77** | **18** | **Quan** |
| | | 133 | 105 | 8 | 73 |
| | | 147 | 105 | 6 | 100 |
| γ-Elemene | **39.88** | **93** | **77** | **12** | **Quan** |
| | | 67 | 41 | 12 | 20 |
| | | 121 | 93 | 6 | 65 |
| | | 105 | 77 | 16 | 35 |
| β-Santalene | **40.10** | **94** | **77** | **20** | **Quan** |
| | | 94 | 79 | 6 | 46 |
| | | 122 | 94 | 4 | 38 |
| Guaia-6,9-diene | **40.37** | **105** | **77** | **16** | **Quan** |
| | | 91 | 65 | 14 | 45 |
| | | 119 | 91 | 12 | 65 |
| | | 161 | 105 | 6 | 55 |
| $C_{15}H_{24}$-204 (69/91/105/161) | **41.05** | **69** | **41** | **6** | **Quan** |
| | | 91 | 65 | 16 | 78 |
| | | 105 | 77 | 16 | 68 |
| | | 161 | 105 | 8 | 45 |
| $C_{15}H_{24}$-204 (91/105/161) | **41.12** | **91** | **65** | **16** | **Quan** |
| | | 105 | 77 | 16 | 90 |
| | | 133 | 105 | 8 | 75 |
| | | 161 | 105 | 6 | 60 |
| $C_{15}H_{24}$-204 (161/105/133/91) | **41.22** | **133** | **105** | **8** | **Quan** |
| | | 91 | 65 | 16 | 50 |
| | | 105 | 77 | 16 | 51 |
| | | 161 | 105 | 8 | 51 |
| $C_{15}H_{24}$-204 (105/91/133/161/189) | **41.30** | **133** | **105** | **8** | **Quan** |
| | | 105 | 77 | 16 | 85 |
| | | 161 | 105 | 8 | 55 |
| | | 189 | 133 | 8 | 15 |
| Acoradiene | **41.70** | **119** | **91** | **12** | **Quan** |
| | | 105 | 77 | 16 | 50 |

| | | | | | |
|---|---|---|---|---|---|
| | | 93 | 77 | 12 | 100 |
| | | 79 | 77 | 10 | 60 |
| $C_{15}H_{24}$-204(105)-1 | **42.04** | **105** | **77** | **16** | **Quan** |
| | | 79 | 77 | 10 | 60 |
| | | 91 | 65 | 16 | 40 |
| | | 105 | 79 | 10 | 45 |
| $C_{15}H_{24}$-204 (189/133)-1 | **42.16** | **133** | **105** | **8** | **Quan** |
| | | 105 | 77 | 18 | 51 |
| | | 189 | 133 | 18 | 31 |
| γ-Curcumene | **42.16** | **119** | **91** | **12** | **Quan** |
| | | 93 | 77 | 10 | 105 |
| | | 105 | 77 | 16 | 65 |
| | | 121 | 93 | 6 | 66 |
| Sesquisabinene | **42.30** | **69** | **41** | **6** | **Quan** |
| | | 41 | 39 | 8 | 24 |
| | | 69 | 39 | 20 | 31 |
| | | 93 | 77 | 12 | 73 |
| γ-Muurelene | **42.79** | **161** | **105** | **10** | **Quan** |
| | | 79 | 77 | 10 | 85 |
| | | 119 | 91 | 12 | 80 |
| | | 161 | 81 | 10 | 39 |
| α-Amorphene | **42.92** | **105** | **77** | **18** | **Quan** |
| | | 119 | 91 | 14 | 72 |
| | | 161 | 105 | 8 | 65 |
| Aristolochene | **42.78** | **105** | **77** | **18** | **Quan** |
| | | 91 | 65 | 16 | 48 |
| | | 189 | 133 | 8 | 23 |
| Germacrene D | **43.29** | **105** | **77** | **16** | **Quan** |
| | | 120 | 105 | 6 | 20 |
| | | 161 | 105 | 8 | 80 |
| $C_{15}H_{24}$-204 (189/133)-2 | **43.41** | **133** | **105** | **8** | **Quan** |
| | | 105 | 77 | 16 | 30 |
| | | 189 | 133 | 8 | 64 |
| $C_{15}H_{24}$-204 (119/93/161) | **43.50** | **119** | **91** | **14** | **Quan** |
| | | 133 | 105 | 8 | 53 |
| | | 161 | 105 | 10 | 42 |
| | | 204 | 161 | 8 | 50 |
| α-Selinene | **43.57** | **105** | **77** | **16** | **Quan** |
| | | 67 | 41 | 12 | 54 |
| | | 81 | 79 | 6 | 86 |
| | | 133 | 105 | 8 | 90 |
| β-Selinene | **43.77** | **133** | **105** | **8** | **Quan** |

| | | | | | |
|---|---|---|---|---|---|
| | | 81 | 79 | 6 | 65 |
| | | 189 | 133 | 8 | 72 |
| α-Farnesene | **43.86** | **123** | **81** | **10** | **Quan** |
| | | 55 | 29 | 8 | 72 |
| | | 107 | 91 | 8 | 245 |
| β-Bisabolene | **45.86** | **69** | **41** | **6** | **Quan** |
| | | 41 | 39 | 6 | 91 |
| | | 69 | 39 | 16 | 33 |
| | | 93 | 51 | 24 | 34 |
| δ-Guaiene | **44.40** | 108 | 93 | 8 | **Quan** |
| | | 79 | 77 | 10 | 35 |
| | | 105 | 77 | 18 | 50 |
| | | 107 | 79 | 6 | 91 |
| β-Cadinene- $C_{15}H_{24}$-204 (119/161/105/134) | **44.55** | **119** | **91** | **12** | **Quan** |
| | | 105 | 79 | 10 | 32 |
| | | 134 | 119 | 6 | 50 |
| | | 161 | 119 | 8 | 28 |
| Dihydroagarofuran | **44.76** | **41** | **39** | **8** | **Quan** |
| | | 109 | 67 | 6 | 80 |
| | | 137 | 94 | 4 | 35 |
| | | 207 | 105 | 12 | 33 |
| $C_{15}H_{24}$-204 (similar to Germarcene B) | **44.81** | **121** | **93** | **6** | **Quan** |
| | | 93 | 77 | 12 | 110 |
| | | 105 | 77 | 16 | 65 |
| | | 147 | 105 | 10 | 27 |
| Sesquicineole | **44.95** | **139** | **43** | **10** | **Quan** |
| | | 43 | 41 | 4 | 15 |
| | | 95 | 55 | 12 | 35 |
| | | 139 | 95 | 8 | 55 |
| Eremophilene | **45.05** | **161** | **119** | **8** | **Quan** |
| | | 105 | 77 | 16 | 110 |
| | | 147 | 105 | 10 | 70 |
| | | 161 | 105 | 10 | 98 |
| β-Sesquiphellandrene | **45.28** | **69** | **41** | **6** | **Quan** |
| | | 41 | 39 | 8 | 36 |
| | | 93 | 77 | 10 | 70 |
| γ-Cadinene | **45.28** | **161** | **105** | **6** | **Quan** |
| | | 79 | 77 | 10 | 65 |
| | | 119 | 91 | 8 | 90 |
| | | 161 | 119 | 6 | 65 |
| δ-Cadinene | **45.45** | **119** | **91** | **10** | **Quan** |
| | | 134 | 119 | 6 | 100 |

| | | | | | |
|---|---|---|---|---|---|
| | | 161 | 105 | 8 | 80 |
| $C_{15}H_{24}$-204 (105) - 2 | **45.64** | **105** | **77** | **16** | **Quan** |
| | | 107 | 91 | 8 | 75 |
| | | 119 | 91 | 10 | 55 |
| | | 147 | 91 | 12 | 55 |
| α-Panasinsene | **46.06** | **122** | **107** | **6** | **Quan** |
| | | 107 | 79 | 8 | 20 |
| | | 122 | 91 | 18 | 25 |
| *trans*-α-Bisabolene | **46.55** | **93** | **77** | **12** | **Quan** |
| | | 67 | 41 | 10 | 20 |
| | | 119 | 91 | 14 | 9 |
| | | 121 | 93 | 6 | 15 |
| Selina-3,7(11)-diene | **47.00** | **107** | **91** | **10** | **Quan** |
| | | 133 | 105 | 8 | 84 |
| | | 161 | 105 | 10 | 70 |
| | | 204 | 161 | 5 | 85 |
| Germacrene B | **48.48** | **121** | **93** | **6** | **Quan** |
| | | 67 | 41 | 12 | 42 |
| | | 107 | 91 | 10 | 84 |
| α-epi-7-epi-5-Eudesmol | **52.58** | **109** | **67** | **10** | **Quan** |
| | | 59 | 31 | 12 | 50 |
| | | 149 | 93 | 8 | 30 |
| $C_{15}H_{26}O$-222 (similar to γ-Eudesmol) | **53.58** | **133** | **105** | **8** | **Quan** |
| | | 59 | 31 | 18 | 9 |
| | | 161 | 105 | 12 | 49 |
| | | 189 | 133 | 8 | 60 |
| Selin-6-en-4α-ol | **54.02** | **81** | **79** | **8** | **Quan** |
| | | 43 | 41 | 4 | 15 |
| | | 105 | 77 | 16 | 70 |
| | | 161 | 105 | 6 | 82 |
| γ-Eudesmol | **54.29** | **133** | **105** | **8** | **Quan** |
| | | 59 | 31 | 12 | 16 |
| | | 161 | 105 | 10 | 55 |
| | | 189 | 133 | 6 | 68 |
| Hinesol | **54.40** | **161** | **105** | **12** | **Quan** |
| | | 59 | 31 | 10 | 50 |
| | | 105 | 77 | 18 | 80 |
| | | 119 | 91 | 10 | 65 |
| $C_{15}H_{26}O$-222 (105/161/59)-1 | **54.58** | **105** | **77** | **18** | **Quan** |
| | | 59 | 31 | 10 | 18 |
| | | 147 | 105 | 6 | 18 |
| | | 161 | 105 | 6 | 105 |

| | | | | | |
|---|---|---|---|---|---|
| Agarospirol | **54.80** | **119** | **91** | **10** | **Quan** |
| | | 59 | 31 | 10 | 70 |
| | | 105 | 77 | 16 | 20 |
| | | 161 | 105 | 12 | 70 |
| $C_{15}H_{26}O$-222 (105/161/59)-2 | **55.29** | **105** | **77** | **18** | **Quan** |
| | | 59 | 31 | 10 | 61 |
| | | 147 | 91 | 8 | 63 |
| | | 161 | 105 | 6 | 120 |
| $C_{15}H_{26}O$-222 (59/81/107/149/161) | **55.42** | **59** | **31** | **12** | **Quan** |
| | | 81 | 79 | 8 | 130 |
| | | 107 | 91 | 6 | 115 |
| | | 149 | 93 | 8 | 50 |
| α-Eudesmol | **56.35** | **59** | **31** | **12** | **Quan** |
| | | 149 | 107 | 8 | 83 |
| | | 161 | 105 | 10 | 140 |
| 7-epi-α-Eudesmol | **56.64** | **122** | **107** | **6** | **Quan** |
| | | 59 | 31 | 10 | 14 |
| | | 107 | 91 | 12 | 55 |
| | | 161 | 105 | 10 | 38 |
| Bulnesol | **57.00** | **107** | **91** | **10** | **Quan** |
| | | 59 | 41 | 8 | 7 |
| | | 135 | 107 | 6 | 70 |

RT, retention times; Precursor I, precursor ions mass to charge; Product I, product ions mass to charge; CE, Collison Energy in electron volt; Quan, ratio quantification - the first transition on each row was used for quantification and the rest for qualification.

**Table S4. List of identified terpenoids without commercially available standards (based on the NIST library and RIs)**

| Compound name | Formula | MW [m/z] | RT [min] | NIST Match | NIST R. Match | $RI_{cal}$ | $RI_{lit}$ | $\Delta_{RIcal-RIlit}$ |
|---|---|---|---|---|---|---|---|---|
| β-Phellandrene | $C_{10}H_{16}$ | 136 | 14.50 | 896 | 903 | 983 | 1021 | 38 |
| $C_{10}H_{18}O$-154 (93/79/121)-1 | $C_{10}H_{18}O$ | 154 | 24.10 | - | - | 1104 | - | - |
| $C_{10}H_{18}O$-154 (93/79/121)-2 | $C_{10}H_{18}O$ | 154 | 26.20 | - | - | 1134 | - | - |
| α-Cubebene | $C_{15}H_{24}$ | 204 | 34.62 | 915 | 925 | 1290 | 1351 | 61 |
| Isoledene * | $C_{15}H_{24}$ | 204 | 35.72 | 921 | 928 | 1311 | 1375 | 64 |
| Cyclosativene * | $C_{15}H_{24}$ | 204 | 35.66 | 936 | 947 | 1313 | 1368 | 55 |
| Ylangene | $C_{15}H_{24}$ | 204 | 35.91 | 899 | 910 | 1318 | 1372 | 54 |
| α-Copaene | $C_{15}H_{24}$ | 204 | 36.22 | 934 | 945 | 1325 | 1376 | 51 |
| 7-epi-Sesquithujene | $C_{15}H_{24}$ | 204 | 36.83 | 900 | 929 | 1335 | 1391 | 56 |
| $C_{15}H_{24}$-204 (105/(120+119)/161) | $C_{15}H_{24}$ | 204 | 36.84 | - | - | 1335 | - | - |
| Sativene* | $C_{15}H_{24}$ | 204 | 37.47 | 936 | 925 | 1348 | 1396 | 48 |
| β-Cubebene | $C_{15}H_{24}$ | 204 | 37.60 | 847 | 884 | 1351 | 1389 | 38 |
| Sesquithujene | $C_{15}H_{24}$ | 204 | 37.60 | 914 | 940 | 1351 | 1402 | 51 |
| β-Isocomene | $C_{15}H_{24}$ | 204 | 37.90 | 818 | 843 | 1358 | 1412 | 54 |
| α-Santalene | $C_{15}H_{24}$ | 204 | 38.45 | 914 | 923 | 1365 | 1420 | 55 |
| *cis*-α-Bergamotene | $C_{15}H_{24}$ | 204 | 38.53 | 925 | 934 | 1366 | 1415 | 49 |
| *trans*-α-Bergamotene | $C_{15}H_{24}$ | 204 | 39.10 | 915 | 934 | 1380 | 1435 | 55 |
| α-Guaiene | $C_{15}H_{24}$ | 204 | 39.69 | 922 | 933 | 1391 | 1439 | 48 |
| γ-Elemene | $C_{15}H_{24}$ | 204 | 39.89 | 923 | 926 | 1395 | 1433 | 38 |
| β-Santalene | $C_{15}H_{24}$ | 204 | 40.20 | 935 | 946 | 1407 | 1448 | 41 |
| Guaia-6,9-diene | $C_{15}H_{24}$ | 204 | 40.37 | 936 | 939 | 1407 | 1443 | 36 |
| $C_{15}H_{24}$-204 (69/91/105/161) | $C_{15}H_{24}$ | 204 | 41.05 | - | - | 1415 | - | - |
| $C_{15}H_{24}$-204 (91/105/161) | $C_{15}H_{24}$ | 204 | 41.12 | - | - | 1417 | - | - |
| $C_{15}H_{24}$-204 (161/105/133/91) | $C_{15}H_{24}$ | 204 | 41.22 | - | - | 1420 | - | - |
| $C_{15}H_{24}$-204 (105/91/133/161/189) | $C_{15}H_{24}$ | 204 | 41.30 | - | - | 1422 | - | - |
| Acoradiene | $C_{15}H_{24}$ | 204 | 41.80 | 900 | 905 | 1436 | 1471 | 36 |
| $C_{15}H_{24}$-204 (105) - 1 | $C_{15}H_{24}$ | 204 | 42.04 | - | - | 1439 | - | - |
| γ-Curcumene | $C_{15}H_{24}$ | 204 | 42.15 | 880 | 904 | 1443 | 1480 | 37 |
| $C_{15}H_{24}$-204 (189/133)-1 | $C_{15}H_{24}$ | 204 | 42.16 | - | - | 1443 | - | - |
| Sesquisabinene | $C_{15}H_{24}$ | 204 | 42.27 | 889 | 894 | 1449 | 1464 | 17 |
| γ-Muurolene | $C_{15}H_{24}$ | 204 | 42.79 | 939 | 944 | 1449 | 1477 | 27 |
| α-Amorphene | $C_{15}H_{24}$ | 204 | 42.93 | 909 | 934 | 1453 | 1482 | 29 |
| Aristolochene | $C_{15}H_{24}$ | 204 | 42.78 | 930 | 938 | 1458 | 1487 | 29 |
| Germacrene D | $C_{15}H_{24}$ | 204 | 43.29 | 900 | 920 | 1459 | 1481 | 21 |
| $C_{15}H_{24}$-204 (189/133)-2 | $C_{15}H_{24}$ | 204 | 43.41 | - | - | 1464 | - | - |
| $C_{15}H_{24}$-204 (119/93/161) | $C_{15}H_{24}$ | 204 | 43.50 | - | - | 1465 | - | - |
| α-Selinene | $C_{15}H_{24}$ | 204 | 43.57 | 950 | 956 | 1468 | 1486 | 18 |
| β-Selinene | $C_{15}H_{24}$ | 204 | 43.77 | 900 | 910 | 1474 | 1494 | 20 |
| α-Farnesene | $C_{15}H_{24}$ | 204 | 43.86 | 962 | 967 | 1476 | 1508 | 32 |
| β-Bisabolene | $C_{15}H_{24}$ | 204 | 43.86 | 948 | 948 | 1476 | 1509 | 33 |
| δ-Guaiene | $C_{15}H_{24}$ | 204 | 44.40 | 927 | 928 | 1480 | 1509 | 29 |
| β-Cadinene- $C_{15}H_{24}$-204(119/161/105/134) | $C_{15}H_{24}$ | 204 | 44.55 | 912 | 900 | 1486 | 1513 | 27 |
| Dihydroagarofuran | $C_{15}H_{26}O$ | 222 | 44.76 | 935 | 950 | 1490 | 1496 | 6 |
| $C_{15}H_{24}$-204 (similar to Germarcene B) | $C_{15}H_{24}$ | 204 | 44.81 | - | - | 1491 | - | - |
| Sesquicineole | $C_{15}H_{26}O$ | 222 | 44.95 | 924 | 912 | 1496 | 1516 | 20 |
| Eremophilene | $C_{15}H_{24}$ | 204 | 45.05 | 842 | 852 | 1497 | 1499 | 2 |
| β-Sesquiphellandrene | $C_{15}H_{24}$ | 204 | 45.20 | 942 | 932 | 1501 | 1524 | 23 |
| γ-Cadinene | $C_{15}H_{24}$ | 204 | 45.28 | 918 | 925 | 1499 | 1513 | 14 |
| δ-Cadinene | $C_{15}H_{24}$ | 204 | 45.45 | 879 | 897 | 1502 | 1524 | 22 |
| $C_{15}H_{24}$-204 (105)-2 | $C_{15}H_{24}$ | 204 | 45.64 | - | - | 1506 | - | - |
| α-Panasinsene | $C_{15}H_{24}$ | 204 | 46.06 | 848 | 857 | 1513 | 1527 | 14 |
| *trans*-α-Bisabolene | $C_{15}H_{24}$ | 204 | 46.55 | 913 | 952 | 1515 | 1512 | -3 |
| Selina-3,7(11)-diene | $C_{15}H_{24}$ | 204 | 47.00 | 937 | 941 | 1520 | 1542 | 22 |

| | | | | | | | | |
|---|---|---|---|---|---|---|---|---|
| Germacrene B | $C_{15}H_{24}$ | 204 | 48.48 | 911 | 913 | 1548 | 1557 | 9 |
| α-epi-7-epi-5-Eudesmol | $C_{15}H_{26}O$ | 222 | 52.58 | 896 | 912 | 1626 | 1616 | -10 |
| $C_{15}H_{26}O$-222 (similar to γ-Eudesmol) | $C_{15}H_{26}O$ | 222 | 53.58 | - | - | 1642 | - | - |
| Selin-6-en-α-ol | $C_{15}H_{26}O$ | 222 | 54.02 | 870 | 896 | 1650 | 1636 | -14 |
| γ-Eudesmol | $C_{15}H_{26}O$ | 222 | 54.29 | 933 | 934 | 1655 | 1631 | -24 |
| Hinesol | $C_{15}H_{26}O$ | 222 | 54.40 | 879 | 917 | 1657 | 1635 | -22 |
| $C_{15}H_{26}O$-222 (105/161/59)-1 | $C_{15}H_{26}O$ | 222 | 54.58 | - | - | 1660 | - | - |
| Agarospirol | $C_{15}H_{26}O$ | 222 | 54.80 | 864 | 926 | 1663 | 1645 | -18 |
| $C_{15}H_{26}O$-222 (105/161/59)-2 | $C_{15}H_{26}O$ | 222 | 55.29 | - | - | 1672 | - | - |
| $C_{15}H_{26}O$-222 (59/81/107/149/161) | $C_{15}H_{26}O$ | 222 | 55.42 | - | - | 1673 | - | - |
| α-Eudesmol | $C_{15}H_{26}O$ | 222 | 56.35 | 900 | 917 | 1675 | 1653 | -22 |
| β-Eudesmol* | $C_{15}H_{26}O$ | 222 | 56.51 | 932 | 940 | 1678 | 1649 | -29 |
| 7-epi-α-Eudesmol | $C_{15}H_{26}O$ | 222 | 56.64 | 859 | 879 | 1687 | 1658 | -29 |
| Bulnesol | $C_{15}H_{26}O$ | 222 | 57.00 | 890 | 907 | 1692 | 1667 | -25 |

*STD of the terpenoids used as reference for illustration of the acceptable difference [$\Delta_{RIcal-RIlit}$] between calculated retention time indexes [$RI_{cal}$] and their literature values [$RI_{lit}$] in the present chromatographical conditions.

**Table S5. Maximum limits of detection (MLOD) of terpenoids**

| Terpenoid | MLOD [ppm] |
|---|---|
| Selina-3,7(11)-diene (as Valencene) | 1267 |
| Guaiol | 2099 |
| Caryophyllene oxide | 1890 |
| C15H26O-222 (similar to γ-Eudesmol) (as Guaiol) | 2099 |
| γ-Eudesmol (as β-Eudesmol) | 1488 |
| α-Eudesmol (as β-Eudesmol) | 1488 |
| β-Eudesmol | 1488 |
| α-Bisabolol | 3391 |

## A Monoterpenoids

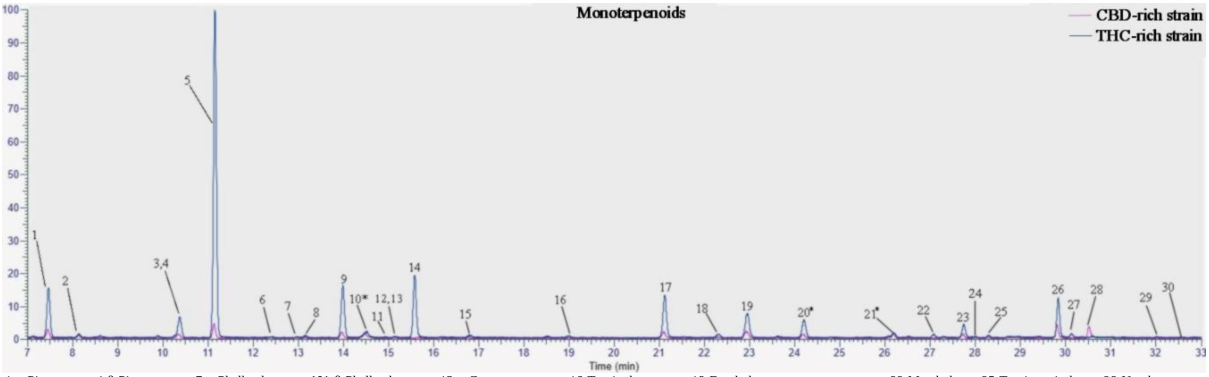


1-α-Pinene
2-Camphene
3-Sabinene
4-β-Pinene
5-β-Myrcene
6-3δ-Carene
7-α-Phellandrene
8-α-Terpinene
9-Limonene
10*-β-Phellandrene
11-*cis*-Ocimene
12-Eucalyptol
13-p-Cymene
14-*trans*-Ocimene
15-γ-Terpinene
16-Terpinolene
17-Linalool
18-Fenchone
19-Fenchol
20*-C10H18O-154 (93/79/121)-1
21*-C10H18O-154 (93/79/121)-2
22-Menthol
23-Borneol
24-Camphor
25-Terpinen-4-ol
26-α-Terpineol
27-Citronellol
28-Nerol
29-Geraniol
30-Bornyl acetate

## B Sesquiterpenoids

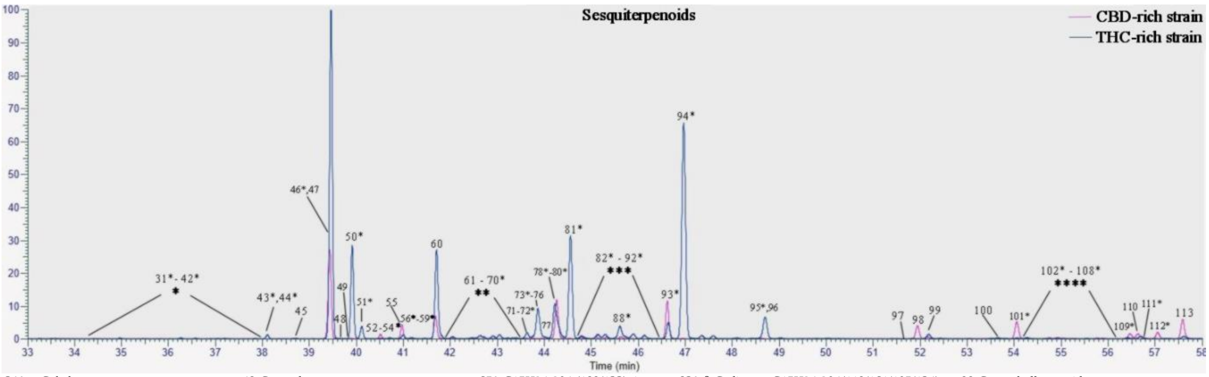


31*-α-Cubebene
32-Isoledene
33-Cyclosativene
34*-Ylangene
35*-α-Copaene
36-α-Funedrene
37*-7-epi-Sesquithujene
38*-C15H24-204 (120 (119+105)/161)
39-Sativene
40*-β-Cubebene
41*-Sesquithujene
42*-β-Isocomene
43*-α-Santalene
44*-*cis*-α-Bergamotene
45-α-Cedrene
46*-*trans*-α-Bergamotene
47-β-Caryophyllene
48-Geranyl acetate
49-β-Cedrene
50*-α-Guaiene
51*-γ-Elemene
52-Aromadendrene
53*-β-Santalene
54*-Guaia-6,9-diene
55-*trans*-β-Farnesene
56*-C15H24-204 (69/91/105/161)
57*-C15H24-204 (91/105/161)
58*-C15H24-204 (161/105/133/91)
59*-C15H24-204 (105/91/133/161/189)
60-α-Humulene
61-Alloaromadendrene
62*-Acoradiene
63*-C15H24-204 (105) - 1
64*-γ-Curcumene
65*-C15H24-204 (189/133)-1
66*-Sesquisabinene
67*-γ-Muurelene
68*-α-Amorphene
69*-Aristolochene
70*-Germacrene D
71- β-Chamigrene
72*-C15H24-204 (189/133)-2
73*-C15H24-204 (119/93/161)
74*-α-Selinene
75-Ledene
76-α-Curcumene
77-Valencene
78*-β-Selinene
79*-α-Farnesene
80*-β-Bisabolene
81*-δ-Guaiene
82*-β-Cadinene- C15H24-204(119/161/105/134)
83-β-Curcumene
84*-Dihydroagarofuran
85*-C15H24-204 (similar to Germarcene B)
86*-Sesquicineole
87*-Eremophilene
88*-β-Sesquiphellandrene
89*-γ-Cadinene
90*-δ-Cadinene
91*-C15H24-204 (105)-2
92*-α-Panasinsene
93*-*trans*-α-Bisabolene
94*-Selina-3,7(11)-diene
95*-Germacrene B
96-*trans*-Nerolidol
97-Globulol
98-Guaiol
99-Caryophyllene oxide
100*-α-epi-7-epi-5-Eudesmol
101*-C15H26O-222 (similar to γ-Eudesmol)
102*-Selin-6-en-α-ol
103*-γ-Eudesmol
104*-Hinesol
105*-C15H26O-222 (105/161/59)-1
106*-Agarospirol
107*-C15H26O-222 (105/161/59)-2
108*-C15H26O-222 (59/81/107/149/161)
109*-α-Eudesmol
110-β-Eudesmol
111*-7-epi-α-Eudesmol
112*-Bulnesol
113*-α-Bisabolol

**Figure S1. Terpenoid profiles of CBD- and THC-rich unfertilized strains of *Cannabis*.** Overlaid chromatograms of unfertilized CBD- and THC-rich female plants divided into (**A**) monoterpenoids and (**B**) sesquiterpenoids. Terpenoids are denoted in numbers and the full names appear below the chromatogram. *Terpenoids that were semi-quantified.

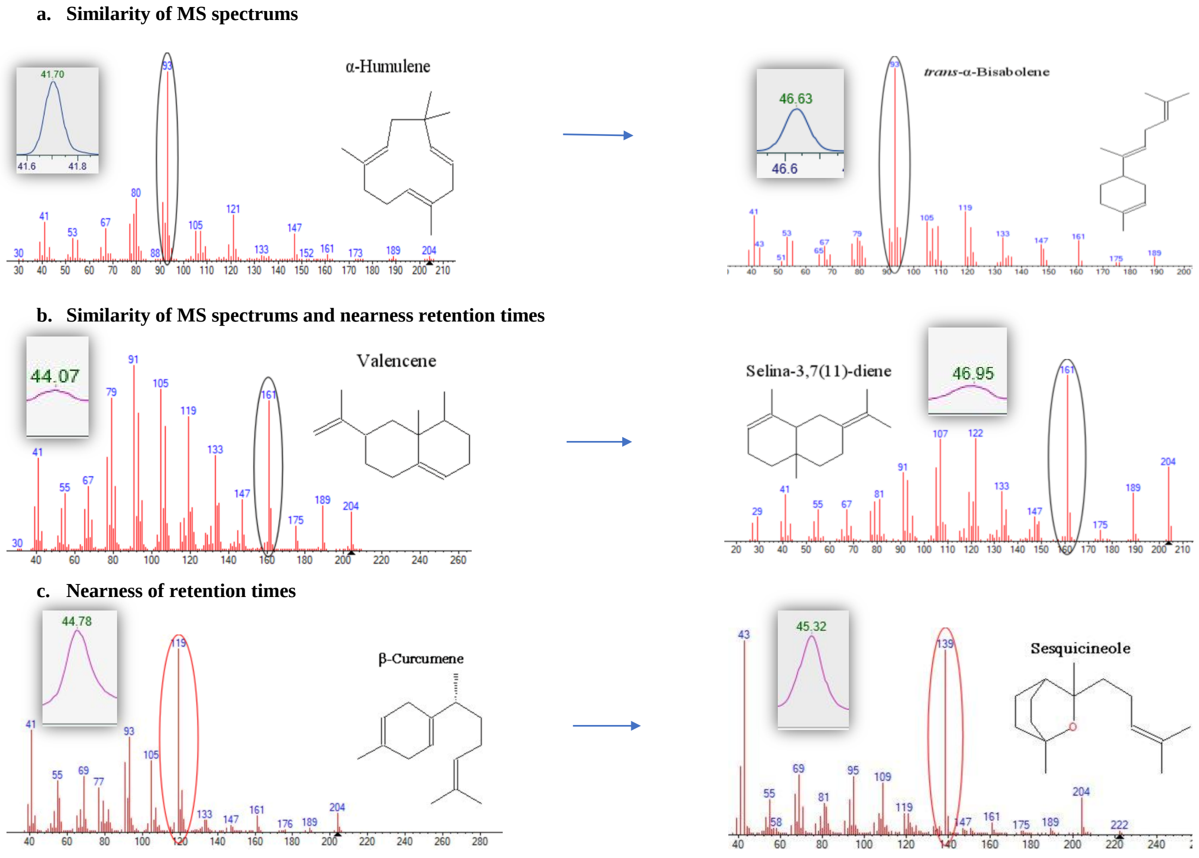


**Figure S2. Principles of terpenoid semi-quantification**. Terpenoids without commercially available analytical standards are semi-quantified according to three principles from more to less fit: (**a**) Similarity of MS spectrums, (**b**) Close similarity of MS spectrums and nearness of retention times, and (**c**) Nearness of retention times only.
